# Prediction Model for Cognitive Impairment among Disabled Older Adults: A Development and Validation Study

**DOI:** 10.3390/healthcare12101028

**Published:** 2024-05-15

**Authors:** Xiangyu Cui, Xiaoyu Zheng, Yun Lu

**Affiliations:** School of International Pharmaceutical Business, China Pharmaceutical University, 639 Longmian Avenue, Jiangning District, Nanjing 211198, China; 3221041112@stu.cpu.edu.cn (X.C.); 3322041367@stu.cpu.edu.cn (X.Z.)

**Keywords:** cognitive impairment, prediction model, nomogram, disabled older adults, machine learning, logistic regression

## Abstract

Disabled older adults exhibited a higher risk for cognitive impairment. Early identification is crucial in alleviating the disease burden. This study aims to develop and validate a prediction model for identifying cognitive impairment among disabled older adults. A total of 2138, 501, and 746 participants were included in the development set and two external validation sets. Logistic regression, support vector machine, random forest, and XGBoost were introduced to develop the prediction model. A nomogram was further established to demonstrate the prediction model directly and vividly. Logistic regression exhibited better predictive performance on the test set with an area under the curve of 0.875. It maintained a high level of precision (0.808), specification (0.788), sensitivity (0.770), and F1-score (0.788) compared with the machine learning models. We further simplified and established a nomogram based on the logistic regression, comprising five variables: age, daily living activities, instrumental activity of daily living, hearing impairment, and visual impairment. The areas under the curve of the nomogram were 0.871, 0.825, and 0.863 in the internal and two external validation sets, respectively. This nomogram effectively identifies the risk of cognitive impairment in disabled older adults.

## 1. Introduction

Cognitive impairment (CI) is a neurodegenerative disorder ranging from mild cognitive impairment (MCI) to dementia. CI primarily characterizes memory decline, orientation dysfunction, and other deteriorations of cognitive function, and it tends to be more prevalent among the older population [1,2,3]. With the accelerated aging process, CI has arisen as a pressing global concern, particularly in low- and middle-income countries [4]. A recent meta-analysis indicated that the global prevalence of MCI is as high as approximately 15.56% [5]. More seriously, 152 million people are expected to be living with dementia globally by 2050 [6]. Notably, around three-quarters of dementia patients have not been definitively diagnosed, which could be 90% in low- and middle-income countries [7]. Identifying high-risk populations for CI based on primary healthcare contexts is an urgent issue globally.

Aging not only affects the decline of cognitive function but also increases the risk of physical disability [8]. Disabled older adults are unable to live independently and are associated with a higher risk of CI [9,10]. Previous investigations demonstrated that the prevalence of CI was higher in disabled older adults [8,11,12]. Moreover, evidence suggested that CI and dementia stood as significant factors in disability among older adults [13]. Disability also accelerated cognitive decline, while both CI and disability were independently associated with all-cause mortality [14,15], further compounding the existing disease burden. Unfortunately, current therapies or medications have limited effectiveness in treating CI [16,17,18]. Given this limitation, effectively identifying high-risk populations early and implementing interventions are crucial for controlling disease progression. Furthermore, disabled older adults often necessitate care from nursing facilities and family members, imposing substantial economic and societal burdens [7]. They are facing challenges in accessing timely medical services and examinations, particularly in developing countries where medical resources are deficient [19]. Therefore, developing an easy-to-use, reliable, and widely used predictive tool is imperative to identify the population at high risk for CI among the disabled population.

Prediction models are commonly employed to estimate the possibility that individuals with specific features will be associated with certain outcomes [20]. In the past five years, several prediction models for identifying CI have been developed in various populations (older adults, middle-aged and older adults, older inpatients, and older adults with hypertension) [21,22,23,24,25,26,27]. However, most of the established models lacked external validation [21,22,23,24,25,27], and the predictors in these models were partly derived from biomarkers [21] or numerous complicated neuropsychological examinations [22], which pose measurement difficulties for disabled older adults relying on community-based healthcare services. Furthermore, heterogeneity exists in risk factors for CI between those influencing the disabled older population and other populations [28,29], and it remains unclear whether previous models can accurately identify CI among disabled older adults. Therefore, developing and validating population-specific prediction models for CI is paramount. Moreover, machine learning (ML) algorithms have emerged as valuable tools in predicting dementia diseases [30]. ML can identify complex relationships between variables from real-world data [31] and perform greater flexibility in handling large datasets. Several prediction models have been constructed for MCI or Alzheimer’s disease by random forest (RF) [3], support vector machines (SVM) [32,33], and extreme gradient boosting (XGBoost) [34] with good performance. However, few studies focused on developing prediction models of CI among disabled older populations and assessing the predictive ability of ML in this group. To address this gap, this study aims to construct and validate a predictive tool for determining CI among the disabled older population. The study will provide a reliable, efficient, and easy-to-use tool to support the screening or early diagnosis of CI in the disabled older population.

## 2. Materials and Methods

This study strictly adhered to the Transparent Reporting of a Multivariable Prediction Model for Individual Prognosis or Diagnosis (TRIPOD) [20].

### 2.1. Data and Participants

We obtained data from the Chinese Longitudinal Healthy Longevity Survey (CLHLS), a comprehensive nationwide survey designed to investigate the health conditions of the older population [35]. The CLHLS has been systematically conducted in eight waves since 1998, covering 23 provinces representing 85% of the Chinese population. The development set was constructed on the eighth wave, which comprised 15,874 participants enrolled between 2017 and 2018. Furthermore, two external validation sets were constructed from the CLHLS conducted during the seventh wave in 2014 and the sixth wave in 2011. These datasets comprised 7192 and 9765 participants, respectively. The CLHLS deployed well-trained investigators to conduct measurements and collect a wide range of information by questionnaires, including demographics, socio-economic information, physical and mental function, and other health-related aspects.

Following previous studies, disabled older adults were assessed using the activities of daily living (ADL) scale across six items: bathing, dressing, toileting, mobility, continence, and eating. Older adults who were partially or completely unable to fulfill at least one of the six items independently were considered disabled [36,37,38].

**Figure 1:**
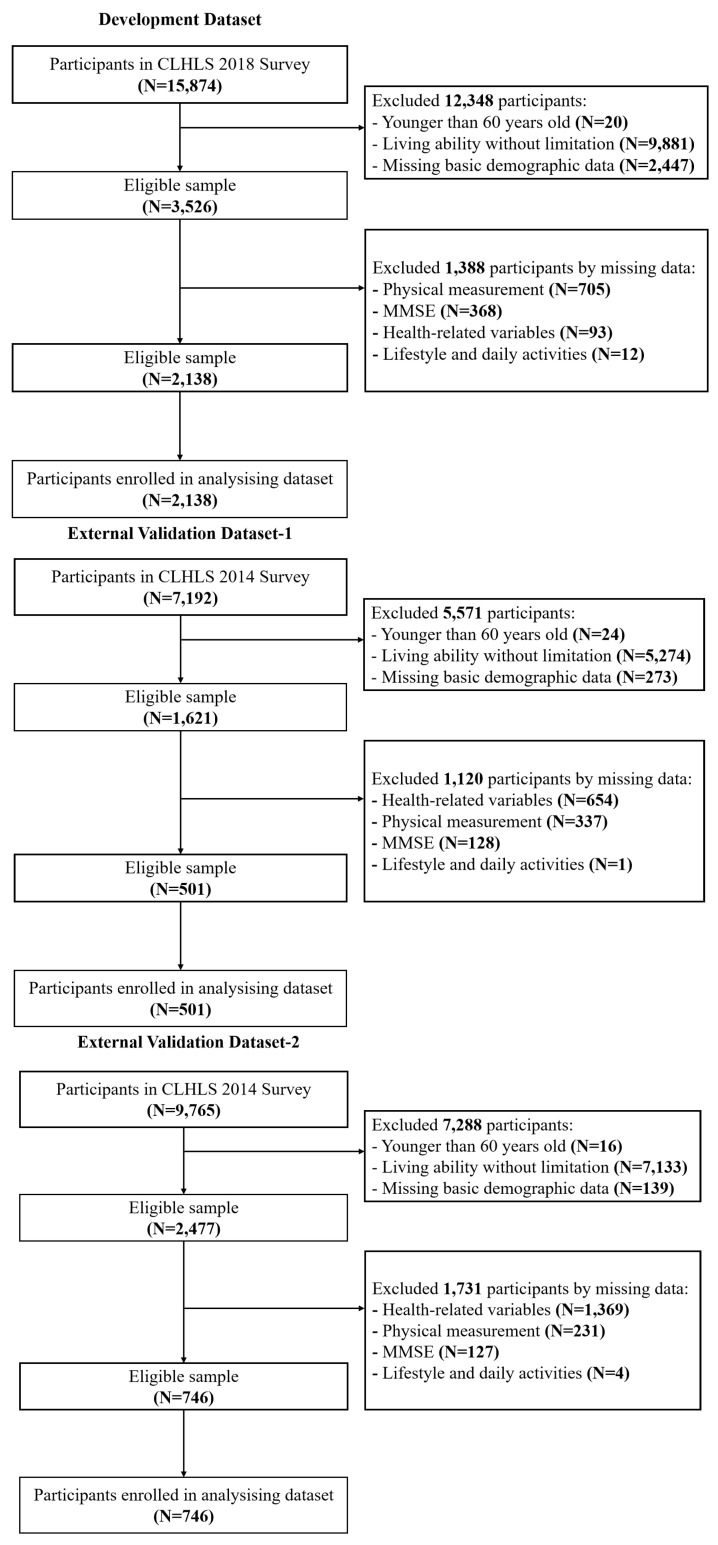
The flowchart of the inclusion and exclusion procedure.

This study’s inclusion criteria were (1) participants aged 60 or older; and (2) participants with ADL disability. We excluded the non-disabled older adults and participants with missing data (Figure 1).

### 2.2. Assessment of Cognitive Impairment

Cognitive function was assessed by the Mini-Mental State Examination (MMSE), with total scores ranging from 0 to 30 [39]. Its reliability and validity have been demonstrated in diagnosing CI and dementia within the Chinese population in clinical and epidemiological studies [40,41,42]. Considering the variations in MMSE scores among older individuals with different educational backgrounds, for participants with no formal education (<1 year), primary education (1–6 years), and higher education (>6 years), cut-off scores of 18, 21, and 25 were established, respectively. These thresholds were determined based on previous studies [18,43].

### 2.3. Candidate Predictors

Socio-demographic variables included age, gender, education level, place of residence, and marital status.

Health-related variables included smoking, drinking, daily exercise, routine medical checkup, kyphosis, ADL scores (scoring 1 for independent completion, 2 for partially dependent completion, and 3 for entirely dependent completion), instrumental activities of daily living (IADL) scores (including: visiting neighbors, shopping, cooking, washing clothes, walking 1 km, lifting 5 kg goods, crouching and standing up three times, and taking public transportation; it was calculated in the same manner as ADL scores), visual impairment (VI) (assessed through the following question: “Can the interviewee see a break in the circle on the cardboard sheet without glasses when lit by a flashlight and distinguish where the break is located?”, the interviewee who was unable to see the given graph and blind was defined as VI [44]), hearing impairment (HI) (assessed through the interviewer to examinate if they could clearly hear what the interviewer was saying, and four options were provided for interviewers: (1) yes, without hearing aids; (2) yes, but need hearing aids; (3) partially hear, despite using hearing aids; (4) cannot hear. Participants identified as having HI included those who could only partially hear despite using hearing aids or could not hear anything at all [44]), wearing hearing aids, chronic diseases (the self-reported question identified six common chronic diseases: “Are you suffering from any of the following diseases which have been identified by doctors” including hypertension, diabetes, heart disease, stroke or cerebrovascular disease, glaucoma, and respiratory diseases), history of falls, wearing dentures, number of natural teeth, tooth cleaning behavior, and childhood famine experiences.

Several variables based on physical measurement were included in the candidate predictors set. Calf circumference (CC), waist circumference (WC), and hip circumference (HC) were measured by trained interviewers using standard methods and tools. Other details can be found on the official website: “https://opendata.pku.edu.cn/dataverse/CHADS” (accessed on 1 November 2022). Additionally, body mass index (BMI), waist-to-hip ratio (WHR), waist-to-height ratio (WHtR), and waist-to-calf ratio (WCR) were calculated to investigate the predictive potential of these variables.

Lifestyle and daily activities variables, including daily housework, garden work, reading newspapers or books, raising domestic animals or pets, playing cards or mah-jongg, and watching TV or listening to the radio, were also incorporated into the candidate predictors set.

### 2.4. Statistical Analyzing

All statistical analyses were performed by R (version 4.2.3). Continuous variables of the development set and external validation sets were presented as means ± standard deviations (SD) or median (interquartile range, IQR), and categorical variables were presented as numbers (proportions). Mean ± SD was used to report normally distributed variables, and median (IQR) was used to report non-normally distributed variables. Fisher’s exact analysis, chi-square tests, t-tests, and the Mann–Whitney U-test were used to assess significant differences (*p*-value < 0.05) between CI and non-CI participants based on the data distribution in the development set. We initially depicted histograms of all continuous variables. When the histograms were skewed or did not clearly show whether the data conformed to a normal distribution, we used the Shapiro–Wilk test to further evaluate normality. To ensure the reliability and precision of our analysis, we assessed the impact of missing data and excluded samples with missing values for any variables. The Least Absolute Shrinkage and Selection Operator (LASSO) regression was used for variable selection in this study. LASSO is a type of regularized regression that effectively addresses the challenges of multicollinearity and overfitting by applying penalty terms to the regression coefficients to encourage model shrinkage of the coefficients. It further excludes variables with high multicollinearity and eliminates variables weakly associated with the dependent variable. The lowest lambda value and corresponding predicting variables were determined using 3-fold cross-validation.

Regarding selecting model-developing methods, logistic regression (LR) is often employed to establish prediction models. ML also demonstrated good performance in the prediction of dementia-related outcomes as well. Therefore, we referred to the recommended ML algorithms in “Guidelines for Developing and Reporting Machine Learning Predictive Models in Biomedical Research: A Multidisciplinary View” [45]. Four algorithms (LR, SVM, RF, and XGBoost) were finally chosen to develop the prediction models. Following previous practices [46,47], the development set was randomly divided into three parts: a 2/3 training set for model training, a 1/6 validation set for parameter tuning, and a 1/6 test set for evaluating the internal predictive performance of different algorithms. Hyperparameters of the machine learning algorithms were fine-tuned using loop statements and grid search on the validation set to balance the performance and generalizability. The four models were comprehensively compared and assessed using AUC, accuracy, precision, specification, sensitivity, and F1-score. We designated AUC as the primary indicator for predictive performance assessment [48].

Additionally, the criteria for evaluating the classification ability of the prediction models were used in this study. An AUC of more than 0.9 was categorized as excellent, 0.8 to 0.9 was categorized as very good, 0.7 to 0.8 as good, and below 0.7 was categorized as poor or not useful [49]. The calibration curve, validated by 1000 bootstrap methods, was used to evaluate the consistency between the predicted probabilities and the results. Decision curve analysis (DCA) was also employed to investigate net benefits. In addition, we reported the R-squared, Akaike information criterion (AIC), and Bayesian information criterion (BIC) for LR to more fully describe the model’s fit. In the modeling process of LR models and ML models construction, CI was used as an objective variable (response variable), and predictors were used as explanatory variables (features). Continuous variables in the predicators as numeric, objective variables, and categorical variables of the predictors as factors were inputted into the model. Quantitative relationships or estimates were calculated by ML algorithms and LR.

A nomogram will be developed for direct application and enhanced convenience if LR exhibits superior predictive performance compared to the other three ML methods. We will use stepwise regression, stopping until the minimum AIC is obtained, and eliminate non-significant variables to minimize biased estimation while maintaining the predictive performance and interpretability of the nomogram. If SVM, XGBoost, or RF is selected to construct the predictive model, the importance ranking of predictors will be calculated and interpreted using Shapley Additive Explanation (SHAP) values.

## 3. Results

### 3.1. Participant Characteristics

In total, 2138 participants were included in the development set, with 1130 (52.9%) identified as CI patients and 1008 (47.1%) as non-CI participants. External validation set-1 (CLHLS 2014) and set-2 (CLHLS 2011) comprised 501 and 746 participants, respectively (Supplementary Table S1). The prevalence of CI for the two datasets was 43.7% and 39.1%, respectively. Statistically significant differences (*p* < 0.05) were observed between the CI and non-CI groups for 31 variables in the development set, including gender, age, place of residence, marital status, education level, ADL score, IADL score, smoking, daily exercise, routine medical checkup, kyphosis, VI, HI, wearing hearing aids, chronic diseases, wearing dentures, number of natural teeth, tooth cleaning behavior, childhood famine experiences, CC, WC, HC, BMI, WHtR, WCR, daily housework, garden work, reading newspapers or books, raising domestic animals or pets, playing cards or mah-jongg, and watching TV or listening to the radio. Drinking (*p* = 0.097), history of falls (*p* = 0.230), and WHR (*p* = 0.165) were examined for non-significant differences (Table 1).

### 3.2. Predictors Selection

**Figure 2:**
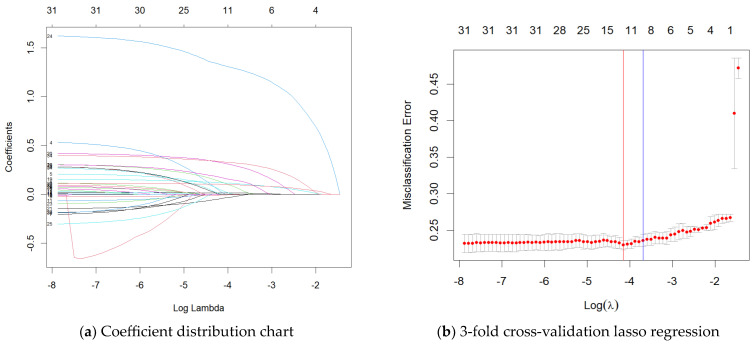
Predictors selected by LASSO regression: (**a**) Coefficient distribution chart. λ is the regularization parameter. As λ increases, the LASSO regression increases the penalty on the coefficients, thus contributing to the possibility that the coefficients of the model variables may be reduced to zero, corresponding to the elimination of certain predictor variables one by one from the figure. (**b**) Three-fold cross-validation LASSO regression. Misclassification error was plotted versus log(λ). The red line is the log(λ) and the corresponding variable for obtaining the minimum value of the misclassification error, and the blue line is the log(λ) one standard error away from the minimum value of the misclassification error. The λ value corresponding to the red line is the optimal regularization parameter, which represents the best model fit and is considered a criterion for determining the combination of variables.

Predictors were selected using LASSO regression and three-fold cross-validation, with the lambda value of 0.016 corresponding to the smallest error. The detailed process is illustrated in Figure 2. From the original 34 variables, a final set of 12 variables was selected, including age, education level, marital status, number of natural teeth, wearing dentures, ADL score, VI, chronic diseases, HI, tooth cleaning behavior, IADL score, and watching TV/listening to the radio.

### 3.3. Model Development and Comparison

Hyperparameters of RF, SVM, and XGBoost were tuned to optimize their performance on the validation set. The default and best parameters were comprehensively reported in Table 2. The hyperparameter combination that demonstrated the best performance in ML algorithms was selected for developing the prediction models.

Based on the results obtained from the test set, LR outperformed other ML methods in terms of predictive performance. It achieved the highest AUC of 0.875 and the highest accuracy of 0.778. Furthermore, LR consistently exhibited a high level of precision (0.808), specificity (0.788), sensitivity (0.770), and F1-score (0.788). After a comprehensive evaluation across different algorithms, LR was chosen as the final prediction model. All the details can be found in Table 3.

### 3.4. Development and Validation of the Nomogram

**Figure 3:**
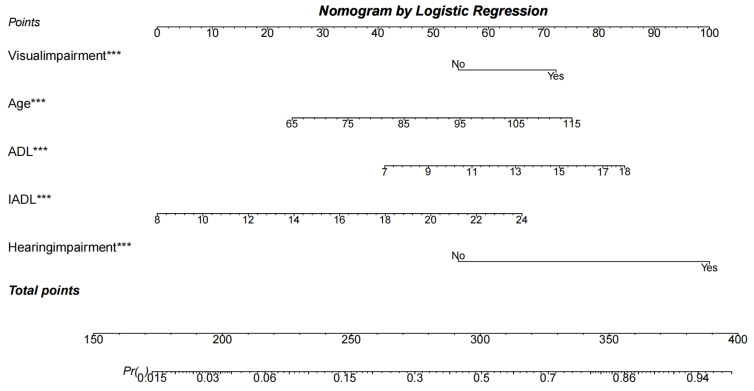
The nomogram developed based on the results of the logistic regression was capable of being scored visually. The nomogram consisted of age (from 65 to 114), VI (yes, no), ADL score (from 7 to 18), IADL score (from 8 to 24), and HI (yes, no). *** indicated *p* < 0.01. (1) The leftmost part of the nomogram is the input variables and corresponds to the categories or values. (2) The category or value of each variable corresponds to the points at the top, and the individual scores corresponding to all variables are accumulated to correspond to the total points at the bottom. (3) The scale at the bottom represents the risk of developing CI, and the risk of CI for that individual can be calculated by making a vertical line across the total points.

A nomogram was developed based on the LR results. Stepwise LR was employed, achieving an AIC of 1417, and non-significant variables were subsequently excluded to further simplify the nomogram. The BIC of the nomogram was 1490 and the R-squared was 0.475. The final nomogram comprised five predictors: age, ADL score, IADL score, HI, and VI (Figure 3). Increasing age (OR = 1.034, 95% CI = 1.016–1.051, *p* < 0.001), ADL score (OR = 1.138, 95% CI = 1.084–1.195, *p* < 0.001), IADL score (OR = 1.145, 95% CI = 1.095–1.197, *p* < 0.001), HI (OR = 4.434, 95% CI = 3.411–5.760, *p* < 0.001), and VI (OR = 1.785, 95% CI = 1.370–2.326, *p* < 0.001) demonstrated to be associated with higher odds for CI. Complete details are provided in Table 4.

**Figure 4:**
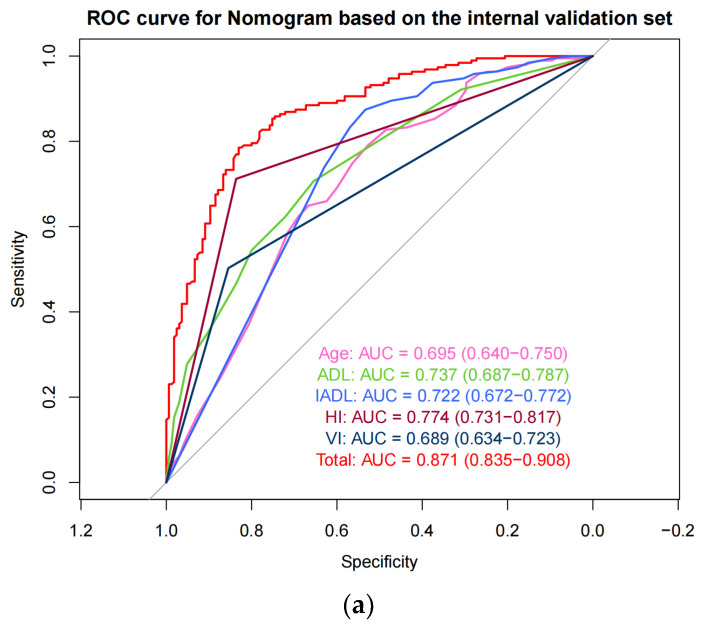

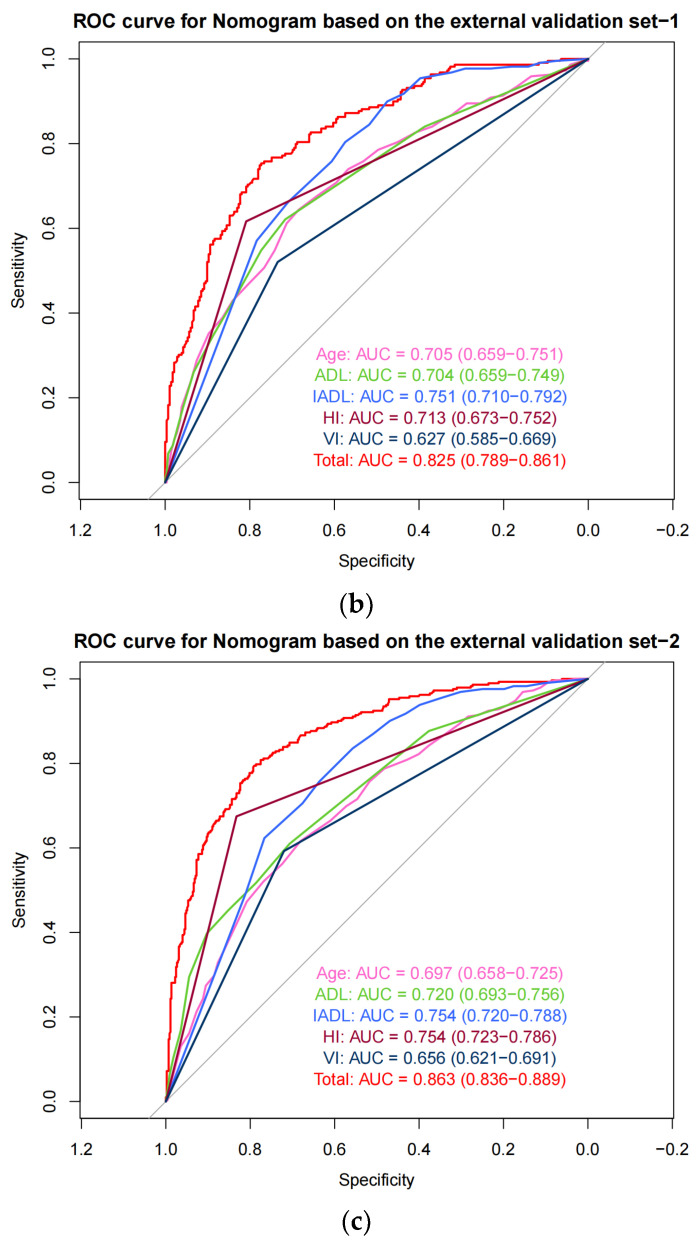
The receiver operating characteristic curve for the nomogram and each predictor in (**a**) the test dataset, (**b**) external validation set-1, and (**c**) external validation set-2. The grey line indicated the receiver operating characteristic curve at an area under the curve of 0.5. ADL: activities of daily living; IADL: instrumental activities of daily living; HI: hearing impairment; VI: visual impairment.

**Figure 5:**
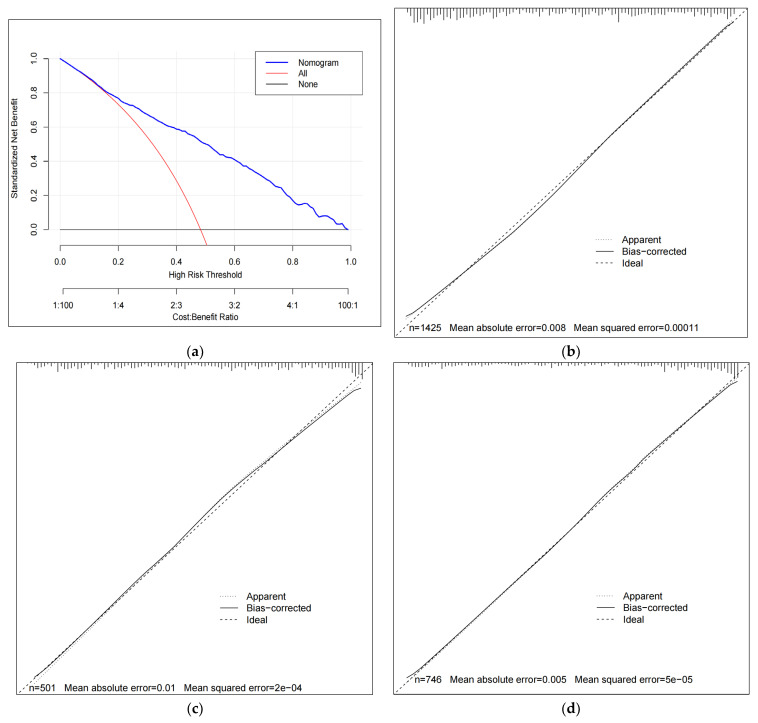
(**a**) Decision curve analysis curve; (**b**) calibration curves based on the test set; (**c**) calibration curves based on the external validation set-1; and (**d**) calibration curves based on the external validation set-2.

We conducted validation using the test set and two external validation sets. The results showed that the nomogram performed well in the internal test set (AUC = 0.871) and showed stable predictive performance in external validation set-1 (AUC = 0.825) and set-2 (AUC = 0.863) (Figure 4). In addition, ROC curves were measured, and AUC values were calculated for each predictor in the three validation sets to further explore the individual predictive ability of these predictors (Figure 4). Moreover, DCA analysis indicated that the nomogram exhibited substantial net benefits across various threshold probabilities from approximately 0.12 to 0.99. The calibration curve displayed excellent performance for predicting CI in internal and external sets (Figure 5).

## 4. Discussion

In this study, we developed and validated a prediction model for identifying the risk of CI in disabled older adults. The predictive performance of LR and three ML algorithms were compared, and we found that LR outperformed. Therefore, a nomogram was further established based on the results of LR comprising five predictors: age, ADL score, IADL score, HI, and VI. These predictors are easily accessible through basic information and simple assessments, which have the advantages of low cost and ease of use.

We evaluated the discriminative ability of the prediction model based on ROC curves and AUC. LR models had stable AUCs of more than 0.8 in all internal and external validation sets, implying “very good” predictive performance [48]. Comparisons with the predictive performance of previous prediction models related to CI constructed in other populations demonstrated that the AUC of the internal validation (AUC = 0.871) set was higher than that of the previous model [21,23,24,25,27] and the model had a wide range of benefits and excellent calibration. This further suggested the validity and reliability of our model in identifying CI among disabled older adults. Among the five predictors, we investigated their independent predictive ability for CI. In the internal validation set, HI demonstrated outstanding predictive ability, followed by ADL, IADL, and age. VI had the weakest predictive ability. In both external validation sets, IADL demonstrated the best predictive ability. In external validation set-2, HI and IADL had comparable AUC, but VI both demonstrated the weakest predictive ability.

Age is an unmodifiable factor and stood as one of the predictors in our study. Our findings supported previous studies. Extensive evidence pointed out that aging is a significant contributor to CI and a primary cause of neurodegenerative disorders [32,50]. A CI predicting study revealed age was an individual predictor, resulting in a C-index of 0.67 [18]. In our study, the AUC for age varied from 0.695 to 0.7 across three validation sets. Notably, most models integrated age as a predictor in models for CI diagnosis [21,22,23,24,25,26]. As a core factor in aging disorders, screening for dementia has been undertaken in multiple countries [51]. Being 75 years of age could be a risk stratification indicator, with evidence suggesting that about 80 percent of individuals with dementia were aged 75 years or older [52]. However, the cost-effectiveness of screening for all ages and of large-scale screening for specific age groups above 75 years remained unclear.

ADL score and IADL score were important predictors in our model. The ADL and IADL scales were the most commonly used to measure the patient’s ability to live independently. These scales reflect the level of disability, with higher scores associated with poorer ability to live independently [53,54]. Dysfunction was significantly associated with cognitive decline in older adults [55,56]. This association may stem from the simultaneous decline in physical and cognitive functioning caused by aging related to changes in brain circuitry and pathology [57,58]. There may also be a bidirectional relationship between somatic functional and cognitive decline [59,60,61,62]. A cohort with an average age of 72 years found that declines in physical function predicted a decline in cognitive function. This association occurred at the same time interval but was not significant at the dissimilar interval [63]. Furthermore, while the concepts of ADL score and IADL score are interrelated, they are not complete substitutes. ADL primarily captures the loss of physical function, whereas IADL is based primarily on psychosocial and executive function [64]. A study on older adults from middle-income countries found that both ADL and IADL were predictors of dementia and MCI. Notably, IADL may have a more sensitive measurement effect [56]. A possible explanation is that the IADL scale involves memory and execution. When these functions are impaired, the IADL can be quickly identified. ADL declines as dementia progresses, eventually affecting even basic activities [65,66]. However, when physical function is poor, IADL may have a floor effect that fails to capture the loss of further daily function and does not accurately predict cognitive function in this population group. Therefore, both ADL and IADL should be included in the prediction model for a comprehensive measurement. The issue of how to slow the progression of disability is essential in the care of the disabled older population [67]. A study conducted among nursing home residents in the United States revealed that disability deterioration exhibited significant heterogeneity across groups with various functional levels. This heterogeneity posed challenges in identifying high-risk populations through progression trajectory analysis [68]. Dynamic features of ADL scores seem to capture the progression of disability. A study of repeated-measurement ADLs found short-term fluctuations in disability, which were significantly associated with mortality. Moreover, ADL fluctuations progressively increased towards the end of life [69]. Targeted care can improve the living quality of people with late-life disability.

VI and HI were predictors of the model and demonstrated strong individual discrimination. VI and HI are components of sensory impairment. Studies have shown that sensory impairment can accelerate cognitive decline and is significantly associated with CI and dementia [70,71]. A cohort study revealed that HI effectively predicted cognitive decline [72]. However, there is no consensus on the mechanism between HI and CI. They were currently explained by the sensory deprivation hypothesis, resource allocation hypothesis, and cognitive load on perception hypothesis [73]. A prospective cohort study found that social participation mediated the association. This could be because eye disease reduces patients’ social activity. Long-term visual impairment impaired their social functioning, affecting the central region of the visual system in the brain [74]. Similar mediators include loneliness and depression [75,76]. Furthermore, a systematic review indicated that the relationship between VI and CI or dementia was similar across studies using different measurements of vision and cognition [77]. This may stem from CI and VI having similar pathologic processes, increasing the risk of CI with aging [78]. Additionally, VI and HI are also significant risk factors in ADL and IADL disabilities, and multiple dysfunctions further exacerbate CI [79]. Notably, improving hearing function has been demonstrated to decrease dementia risk. Hearing restoration devices were associated with a 19 percent lower risk of CI [80]. However, a multicenter randomized controlled trial revealed differences in benefits across populations. Hearing interventions improved cognitive function for only 3 years in an older group with cognitive decline. The effect was insignificant in a group with normal cognitive function [81]. Additionally, screening and improvement of VI have been shown to be effective in public health practice [82]. Vision assessment is a widely accessible, low-cost test. Vision-related training (e.g., visual field training) can also be performed with devices such as mobile phones [83,84].

We compared predictors of CI in older adults with and without disabilities. Previous predictors in CI prediction models for non-disabled older populations include age [21,24,25,26], education level [23,24,25,26,27], gender [25,26], place of residence [25,26], reading books [25,27], physical exercise [23,26,27], smoking [23,27], drinking [23], cardiovascular disease [21,23], and BMI [21]. Predictors in the model developed for the disabled older adults in this study included age, ADL score, IADL score, HI, and VI. We found that predictors in the non-disabled older adult population were mainly socio-demographic factors (e.g., gender, education level, place of residence), and common negative health behaviors and diseases. In contrast, impairments in somatic and sensory functioning predicted CI more sensitively in disabled older adults. This difference may stem from two reasons. First, impairments in sensory functioning are associated with physiological alterations in an individual’s brain function, which have a more direct negative impact [85,86]. Secondly, the social isolation and loneliness resulting from reduced social participation due to impaired ability and independence in daily living significantly contribute to cognitive decline [87]. Preventing further deterioration in physical and sensory function is essential for managing cognitive decline in disabled older adults.

We developed the first model and nomogram for CI among the disabled population, which could be further applied in CI diagnosis. Using nationally representative datasets to develop and validate the model, this study can significantly reduce the selection bias and limitations of single centers and small samples. While ML showed outstanding diagnostic performance in other populations and different types of dementia, LR performed better in disabled older adults. The nomogram based on the LR results can assist in identifying the disabled population at risk during screening for CI. It can also increase the accuracy of the assessment and further improve convenience in public health practice. A review suggested that low- and middle-income countries were most prone to experience the impact of dementia. Public health systems need to introduce early diagnosis and person-centered prevention practices [88]. This model is a reliable tool for primary healthcare institutions to identify the high-risk population with CI by collecting simple examinations and low-cost questionnaires. It applies in rural China and other low- and middle-income areas with poor facilities. Disabled older adults with difficulty completing a standard cognitive function assessment can also request their caregivers to complete it as a proxy. We took an example to illustrate how to use this nomogram. If a disabled older adult who is 75 years old (34 points) with an HI (99 points), no VI (54 points), an ADL score of 16 (76 points), and an IADL score of 22 (58 points) for a total of 321 points, this suggests that the probability of CI is approximately 58%. This case demonstrates that users can easily complete a risk assessment and seek timely medical and nursing services. In addition, China’s long-term care insurance provides coverage for people with disabilities [89]. This insurance will determine cognitive function when assessing whether or not they can be reimbursed for their level of care. The nomogram can effectively help identify the probability of CI to reduce the assessment process, support decision-making, and inform other healthcare policies.

Several limitations need to be acknowledged in this study. First, despite constructing validation groups from internal and external sets, further validation in public health practice needs to confirm the robustness and generalizability. Second, our data were obtained through patient self-reported questionnaires, which relied on participants’ memory for specific questions, potentially introducing recall bias into the study. Third, it is important to note that our model was constructed on Chinese disabled older adults, and its suitability should be assessed by other researchers considering different populations. Despite these limitations, this study holds value in identifying CI among the disabled older population.

## 5. Conclusions

This study developed and validated the first CI prediction model among disabled older adults and further developed a nomogram that was more vivid and convenient for use. With only five low-cost and easily measurable variables included (age, ADL score, IADL score, HI, and VI), this prediction model can effectively predict the risk of CI among the disabled older population, and the information could be easily accessed by questionnaires. This provides a reliable tool for CI screening and diagnosis among the disabled older population within community and primary healthcare contexts, particularly beneficial in low- and middle-income countries and resource-limited areas.

## Figures and Tables

**Table 1:** The characteristics of participants in the development dataset.

Variable	Categories	CI (N = 1130)	Non-CI (N = 1008)	Total (N = 2138)	Statistical Test	*p*-Value
Gender, *n* (%)	Male	327 (29%)	411 (41%)	738 (35%)	χ2 test	<0.001
	Female	803 (71%)	597 (59%)	1400 (65%)		
Age, mean (SD)		97.5 (7.0)	91.9 (9.5)	94.8 (8.7)	*t*-test	<0.001
Place of residence, *n* (%)	Urban	663 (59%)	661 (66%)	1324 (62%)	χ2 test	0.001
	Rural	467 (41%)	347 (34%)	814 (38%)		
Marital status, *n* (%)	Married	114 (10%)	255 (25%)	369 (17%)	χ2 test	<0.001
	Others	1016 (90%)	753 (75%)	1769 (83%)		
Education level, *n* (%)	Absence of formal education (<1 year)	825 (73%)	612 (61%)	1437 (67%)	χ2 test	<0.001
	Primary education (1~6 years)	202 (18%)	250 (25%)	452 (21%)		
	Higher education (over 6 years)	103 (9%)	146 (14%)	249 (12%)		
ADL score, mean (SD)		11.4 (3.3)	9.1 (2.4)	10.4 (3.1)	*t*-test	<0.001
IADL score, mean (SD)		22.8 (2.5)	19.3 (5.0)	21.2 (4.2)	*t*-test	<0.001
Smoking, *n* (%)	No	1056 (93%)	901 (89%)	1957 (92%)	χ2 test	<0.001
	Yes	74 (7%)	107 (11%)	181 (8%)		
Drinking, *n* (%)	No	1050 (93%)	916 (91%)	1966 (92%)	χ2 test	0.097
	Yes	80 (7%)	92 (9%)	172 (8%)		
Daily exercise, *n* (%)	No	1037 (92%)	796 (79%)	1833 (86%)	χ2 test	<0.001
	Yes	93 (8%)	212 (21%)	305 (14%)		
Routine medical checkup, *n* (%)	No	637 (56%)	429 (43%)	1066 (50%)	χ2 test	<0.001
	Yes	493 (44%)	579 (57%)	1072 (50%)		
Kyphosis, *n* (%)	No	438 (39%)	531 (53%)	969 (45%)	χ2 test	<0.001
	Yes	692 (61%)	477 (47%)	1169 (55%)		
VI, *n* (%)	No	509 (45%)	757 (75%)	1266 (59%)	χ2 test	<0.001
	Yes	621 (55%)	251 (25%)	872 (41%)		
HI, *n* (%)	No	338 (30%)	775 (77%)	1113 (52%)	χ2 test	<0.001
	Yes	792 (70%)	233 (23%)	1025 (48%)		
Wearing hearing aids, *n* (%)	No	656 (58%)	724 (72%)	1380 (65%)	χ2 test	<0.001
	Yes	474 (42%)	284 (28%)	758 (35%)		
Chronic diseases, *n* (%)	0	582 (52%)	355 (35%)	937 (44%)	χ2 test	<0.001
	1	313 (28%)	333 (33%)	646 (30%)		
	2	136 (12%)	200 (20%)	336 (16%)		
	≥3	99 (8%)	120 (12%)	219 (10%)		
History of falls, *n* (%)	No	754 (67%)	698 (69%)	1452 (68%)	χ2 test	0.230
	Yes	376 (33%)	310 (31%)	686 (32%)		
Wearing dentures, *n* (%)	No	805 (71%)	566 (56%)	1371 (64%)	χ2 test	<0.001
	Yes	325 (29%)	442 (44%)	767 (36%)		
Number of natural teeth, median (Q1, Q3)		0 (0, 3)	1 (0, 7)	0 (0, 4)	Mann–Whitney U-test	<0.001
Tooth cleaning behavior, *n* (%)	Rarely brush teeth	694 (61%)	398 (39%)	1092 (51%)	χ2 test	<0.001
	Regular toothbrushing	436 (39%)	610 (61%)	1046 (49%)		
Childhood famine experiences, *n* (%)	No	228 (20%)	283 (28%)	511 (24%)	χ2 test	<0.001
	Yes	902 (80%)	725 (72%)	1627 (76%)		
CC (cm), mean (SD)		28.0 (6.7)	30 (6.0)	28.9 (6.5)	*t*-test	<0.001
WC (cm), mean (SD)		80.5 (12.5)	84.3 (12.1)	82.3 (12.4)	*t*-test	<0.001
HC (cm), mean (SD)		87.5 (12.6)	91.1 (11.7)	89.2 (12.3)	*t*-test	<0.001
BMI (kg/m^2^), mean (SD)		18.8 (7.1)	21.1 (5.9)	19.9 (6.7)	*t*-test	<0.001
WHR (%), mean (SD)		0.9 (0.1)	0.9 (0.1)	0.9 (0.1)	*t*-test	0.165
WHtR (%), mean (SD)		0.5 (0.2)	0.5 (0.1)	0.5 (0.1)	*t*-test	<0.001
WCR (%), mean (SD)		3.0 (1.1)	2.9 (0.6)	3.0 (1.0)	*t*-test	0.003
Daily housework, *n* (%)	Always	34 (3%)	129 (13%)	163 (8%)	χ2 test	<0.001
	Sometimes	21 (2%)	91 (9%)	112 (5%)		
	Never	1075 (95%)	788 (78%)	1863 (87%)		
Garden work, *n* (%)	Always	22 (2%)	71 (7%)	93 (4%)	χ2 test	<0.001
	Sometimes	14 (1%)	39 (4%)	53 (3%)		
	Never	1094 (97%)	898 (89%)	1992 (93%)		
Reading newspapers or books, *n* (%)	Always	27 (2%)	102 (10%)	129 (6%)	χ2 test	<0.001
	Sometimes	27 (2%)	62 (6%)	89 (4%)		
	Never	1076 (96%)	844 (84%)	1920 (90%)		
Raising domestic animals or pets, *n* (%)	Always	11 (1%)	48 (5%)	59 (3%)	χ2 test	<0.001
	Sometimes	13 (1%)	29 (3%)	42 (2%)		
	Never	1106 (98%)	931 (92%)	2037 (95%)		
Playing cards or mah-jongg, *n* (%)	Always	10 (1%)	42 (4%)	52 (2%)	χ2 test	<0.001
	Sometimes	20 (2%)	57 (6%)	77 (4%)		
	Never	1100 (97%)	909 (90%)	2009 (94%)		
Watching TV or listening to the radio, *n* (%)	Always	223 (20%)	481 (48%)	704 (33%)	χ2 test	<0.001
	Sometimes	123 (11%)	190 (19%)	313 (15%)		
	Never	784 (69%)	337 (33%)	1121 (52%)		

ADL: activities of daily living; IADL: instrumental activities of daily living; CC: calf circumference; WC: waist circumference; HC: hip circumference; BMI: body mass index; WHR: waist-to-hip ratio; WHtR: waist-to-height ratio; WCR: waist-to-calf ratio.

**Table 2:** Hyperparameters of machine learning models based on validation set.

Algorithms	Parameters	Default Parameters	Optimal Parameters	Area Under the Curve		Accuracy	
				Default Parameters	Optimal Parameters	Default Parameters	Optimal Parameters
RF	ntree	500	800	0.819	0.822	0.747	0.750
	mtry	2	1				
	maxnodes	Default	Default				
	nodesize	1	6				
SVM	cost	1	5	0.772	0.835	0.706	0.753
	gamma	0.5	0.01				
	kernel	RBF	POLY				
	degree	3	4				
	coef0	0	1				
XGBoost	eta	0.3	0.2	0.781	0.824	0.708	0.747
	gamma	0	0.8				
	max_depth	6	9				
	min_child weight	1	1				
	subsample	1	0.85				
	colsample_bytree	1	1				
	nrounds	50	7				

maxnodes: representing the maximum number of nodes for each tree. In the R package “randomForest”, it was not set (“Default”), and trees could unlimitedly grow to the maximum possible; kernel: representing the kernel function of SVM. “RBF” meant radial basis function, and “POLY” meant polynomial kernel function.

**Table 3:** The predictive performance of four algorithms on test set.

Algorithms	AUC	Accuracy	Precision	Specification	Sensitivity	F1-Score
LR	0.875	0.778	0.808	0.788	0.770	0.788
RF	0.829	0.762	0.773	0.735	0.785	0.779
SVM	0.833	0.745	0.768	0.743	0.747	0.757
XGBoost	0.836	0.762	0.826	0.789	0.741	0.781

AUC: area under the curve; LR: logistic regression; RF: random forest; SVM: support vector machine.

**Table 4:** The relationship between predictors and CI in nomogram based on the development dataset by logistic regression.

Variables	β	OR	95% CI	*p*-Value
Age	0.033	1.034	(1.016, 1.051)	<0.001
ADL score	0.129	1.138	(1.084, 1.195)	<0.001
IADL score	0.135	1.145	(1.095, 1.197)	<0.001
HI				
No	Reference	1		
Yes	1.489	4.434	(3.411, 5.760)	<0.001
VI				
No	Reference	1		
Yes	0.580	1.785	(1.370, 2.326)	<0.001
Intercept	−8.265	0.001	(0.001, 0.001)	<0.001

OR: odds ratio; CI: confidence interval; ADL: activities of daily living; IADL: instrumental activities of daily living.

## Data Availability

The data utilized in this study are from the Chinese Longitudinal Healthy Longevity Survey (CLHLS) conducted by Peking University. All the data were openly available. The data results that support the findings of this study are available in “Opendata” with the identifier https://doi.org/10.18170/DVN/WBO7LK.

## Supplementary Materials

The following supporting information can be downloaded at: https://www.mdpi.com/article/10.3390/healthcare12101028/s1, Table S1: The characteristics of external datasets.
