# Supplementary material for: Prediction Model for Cognitive Impairment among Disabled Older Adults: A Development and Validation Study
Source: Healthcare (Basel). 2024 May 15;12(10):1028. doi: 10.3390/healthcare12101028 (PMC11121056; doi:10.3390/healthcare12101028)
Supplement: Supplementary file 1 [file healthcare-12-01028-s001.zip › healthcare-2953363-supplementary.pdf]

Supplementary Table S1. The characteristics of external datasets.

Variable	Categories	Ex-validation set-1 (N=501)	Ex-validation set-2 (N=746)
Gender, n (%)	Male	209 (42%)	303 (41%)
	Female	292 (58%)	443 (59%)
Age, mean (SD)		90.7 (10%)	89.9 (10%)
Place of residence, n (%)	Urban	271 (54%)	447 (60%)
	Rural	230 (46%)	299 (40%)
Marital status, n (%)	Married	138 (28%)	225 (30%)
	Others	363 (72%)	521 (70%)
Education level, n (%)	Absence of formal education (<1 year)	327 (65%)	469 (63%)
	Primary education (1~6 years)	136 (27%)	187 (25%)
	Higher education (over 6 years)	38 (8%)	90 (12%)
ADL score, mean (SD)		9.4 (2.6)	9.3 (2.6)
IADL score, mean (SD)		19.7 (5.1)	19.0 (5.0)
Smoking, n (%)	No	452 (90%)	641 (86%)
	Yes	49 (10%)	105 (14%)
Drinking, n (%)	No	457 (91%)	642 (86%)
	Yes	44 (9%)	104 (14%)
Daily exercise, n (%)	No	405 (81%)	561 (75%)
	Yes	96 (19%)	185 (25%)
Routine medical checkup, n (%)	No	241 (48%)	171 (23%)
	Yes	260 (52%)	575 (77%)
Kyphosis, n (%)	No	211 (42%)	382 (51%)
	Yes	290 (58%)	364 (49%)
VI, n (%)	No	312 (62%)	446 (60%)
	Yes	189 (38%)	300 (40%)
HI, n (%)	No	312 (62%)	473 (63%)
	Yes	189 (38%)	273 (37%)
Wearing hearing aids, n (%)	No	344 (69%)	521 (70%)
	Yes	157 (31%)	225 (30%)
Chronic diseases, n (%)	0	247 (49%)	352 (47%)
	1	153 (31%)	231 (31%)
	2	58 (12%)	101 (14%)
	⩾3	43 (8%)	62 (8%)
Wearing dentures, n (%)	No	356 (71%)	526 (71%)
	Yes	145 (29%)	220 (29%)
Number of natural teeth, median (Q1, Q3)		5 (0, 12)	6 (1, 14)
Tooth cleaning behavior, n (%)	Rarely brush teeth	360 (72%)	471 (63%)
	Regular toothbrushing	141 (28%)	275 (37%)
Childhood famine	No	140 (28%)	171 (23%)

experiences, n (%)	Yes	361 (72%)	575 (77%)
CC (cm), mean (SD)		28.8 (6.4)	NA
WC (cm), mean (SD)		79.9 (13.5)	80.9 (15.3)
HC (cm), mean (SD)		NA	NA
BMI (kg/m^2), mean (SD)		21.4 (4.1)	21.2 (4.6)
WHR (%), mean (SD)		NA	NA
WHtR (%), mean (SD)		0.5 (0.1)	0.5 (0.1)
WCR (%), mean (SD)		2.9 (0.7)	NA
Daily housework, n (%)	Always	81 (16%)	107 (14%)
	Sometimes	34 (7%)	59 (8%)
	Never	386 (77%)	580 (78%)
Garden work, n (%)	Always	43 (9%)	64 (9%)
	Sometimes	15 (3%)	47 (6%)
	Never	443 (88%)	635 (85%)
Reading newspapers or books, n (%)	Always	28 (6%)	58 (8%)
	Sometimes	33 (7%)	45 (6%)
	Never	440 (87%)	643 (86%)
Raising domestic animals or pets, n (%)	Always	36 (7%)	48 (6%)
	Sometimes	12 (2%)	28 (4%)
	Never	453 (91%)	670 (90%)
Playing cards or mah-jongg, n (%)	Always	11 (2%)	18 (2%)
	Sometimes	35 (7%)	40 (5%)
	Never	455 (91%)	688 (93%)
Watching tv or listening to the radio, n (%)	Always	216 (43%)	295 (40%)
	Sometimes	92 (18%)	133 (18%)
	Never	193 (39%)	318 (43%)

* ADL: activities of daily living; IADL: instrumental activities of daily living; CC: Calf circumference; WC: waist circumference; HC: hip circumference; BMI: body mass index; WHR: waist-to-hip ratio; WHtR: waist-to-height ratio; WCR: waist-to-calf ratio; NA: not applicable.
